# High Social Motivation Can Promote Time-Based Prospective Memory Performance

**DOI:** 10.3390/bs15081015

**Published:** 2025-07-26

**Authors:** Yadong Zhou, Mingyuan Wang, Yu Tian, Yunfei Guo

**Affiliations:** 1 Editorial Department of Journal of Hangzhou Normal University (Social Science Edition), Hangzhou Normal University, Hangzhou 310030, China; 2 Faculty of Education, Henan University, Kaifeng 475001, China

**Keywords:** social motivation, intensity, prospective memory, attention, importance

## Abstract

Prospective memory, the ability to remember to execute a preplanned activity, is important in social interactions, insofar as such interactions frequently involve preplanned activities. The importance of prospective memory varies across different social contexts and individuals generally make greater efforts to ensure the completion of prospective memory tasks under high social motivation conditions compared to low social motivation conditions. We aimed to investigate the effects of various levels of social motivation on prospective memory. A single-factor between-subjects experimental design was implemented to explore the influence of social motivation intensity on time-based prospective memory and its processing mechanism. We found that only the high social motivation group demonstrated superior prospective memory performance compared to the control group. The high social motivation group also had slower response speeds in response to the ongoing tasks than both the control group and the low social motivation group, but the number of strategies used was not different among the three groups. Moreover, in comparison with the control group, the high social motivation group monitored time more frequently. The results suggest that only high-intensity social motivation can promote time-based prospective memory performance, and increased attention consumption is necessary for the occurrence of this effect.

## 1. Introduction

The capacity to remember to execute planned actions in a future situation or time is known as prospective memory (Einstein & McDaniel, 1990). Some PM tasks have clear external cues, for example, remembering to hand in a document when you pass by the administrative office, which is event-based prospective memory. Other PM tasks do not have clear external cues, which need to be implemented at a specific time, such as remembering to pick someone up at the airport at 12 o’clock today, which is referred to as time-based prospective memory (TBPM). PM studies conducted in the laboratory usually use a dual-task paradigm, and PM tasks are inserted into ongoing tasks (Hockey & Cutmore, 2021). As basic tasks, ongoing tasks are usually given priority, while PM tasks are easily treated as secondary processing objects (Cohen et al., 2003; Guo et al., 2023). However, in daily life, we often make plans for future actions, and we often plan to help others do something in social situations. PM tasks in such social situations are often considered more important and are readily prioritized (Walter & Meier, 2017). Therefore, the processing mechanism underpinning PM tasks when social motivation is a factor should be different from general PM tasks.

### 1.1. Motivational Cognitive Model, Social Motivation, and Prospective Memory

The motivational cognitive model asserts that PM tasks associated with social motivation are usually regarded as more important (Penningroth & Scott, 2013a), and social motivation can also increase the importance placed on PM tasks (Penningroth et al., 2011). When tasks are considered more important, individuals’ PM performance may be improved in two ways. The first way is to improve the accessibility of PM by using various strategies, which do not cost additional attention during the PM retention and extraction phases (Penningroth & Scott, 2013b). The second way is to encourage people to give PM tasks more attention, which will enhance their PM performance (Penningroth & Scott, 2014). Some studies have examined how social motivation affects event-based prospective memory. For example, Brandimonte et al. (2010) informed the participants of the social motivation group that the study was important for the experimenter’s graduation thesis. They found that social motivation could improve individuals’ event-based prospective memory accuracy, but the attention consumption of participants in the social motivation group on event-based prospective memory was less than that used by those in the control group. In another study, Walter and Meier (2017) further directed social motivation to event-based prospective memory tasks only. Participants in the social motivation group were informed that their successful completion of the event-based prospective memory tasks was important to the experimenter. The results showed that social motivation also improved individuals’ event-based prospective memory performance, and the promotional effect of social motivation did not require additional attention consumption. In summary, the promotional effect of social motivation on event-based prospective memory may depend on the path of automated processing in the motivational cognitive model.

### 1.2. The Processing Mechanism of Social Motivation Promoting Time-Based Prospective Memory

There are differences in the processing mechanisms between event-based prospective memory and TBPM. Event-based prospective memory has clear external cues and is easily improved by adopting several strategies without additional attention consumption. However, the key to the successful execution of TBPM is time estimation (Gan & Guo, 2019), and TBPM needs substantial self-initiated attention resources to process time information (Mioni et al., 2020). Both internal effort and the consideration of external time information may be necessary for the successful completion of TBPM tasks (Hu & Qi, 2018). We refer to the former as internal attention and the latter as external attention (Guo et al., 2022). The results of ongoing tasks typically reflect internal attention, while the performance of checking the time usually reflects external attention (Hu & Qi, 2018). It is not clear in the current research if the promotional effect of social motivation on TBPM is more dependent on attention. If there is a promotional effect of social motivation on TBPM, does it depend more on the pathway of controlled processing? Do participants in the social motivation condition rely more on internal attention or external attention? Verifying these questions will help to clarify the processing mechanism of TBPM promoted by social motivation.

Several researchers have focused on the relationship between social motivation and TBPM specifically. For example, Altgassen et al. (2019) focused on the relationship between social motivation in autistic children and TBPM. Children in the social motivation group were informed that the purpose of the experiment was to determine the specific performance of ongoing tasks in one minute. They found no difference in TBPM performance between the children in the social motivation group and those in the control group. In another study, Altgassen et al. (2010) investigated how older and younger individuals performed TBPM tasks under a social motivation condition compared to a control condition. In the social motivation group, participants were informed that the research was a planning phase, and they wanted to know the number of times participants performed the ongoing tasks within 2 min. They discovered that social motivation increased TBPM accuracy in the elderly, and the social motivation group and the control group did not differ in the ongoing task. The investigators believed that the promotional effect of social motivation was also automated, without attention consumption. However, the manipulation of social motivation has been problematic in existing studies. In these studies, participants were informed that the purpose of the experiment was to determine how many ongoing tasks they could complete each time under the social motivation condition, thus directing social motivation to the ongoing task. This, however, is contrary to the aim of Altgassen et al., and there are also differences in the effects of social motivation on TBPM among different studies and groups. As a result, the above studies by Altgassen and colleagues have not been able to fully verify the motivational cognitive model’s claims.

### 1.3. Social Motivation Intensity and Time-Based Prospective Memory

It is possible that different intensities of social motivation have different effects on TBPM. Compared with low-intensity motivation, individuals with high-intensity motivation usually allocate more attention to PM tasks and have better PM performance (Brandimonte & Ferrante, 2015; Han et al., 2017). Participants were informed by Altgassen et al. (2010, 2019) that they were getting ready for an upcoming experiment with the requirement under the condition of social motivation, which was not very important things to others and it was difficult for the participants to perceive the importance of the task, so the intensity of social motivation should have been relatively low. Participants in the social motivation group were informed by Brandimonte et al. (2010) that the research formed their graduation thesis, which was significantly more important to others, and the intensity of social motivation should have been higher. Although Brandimonte et al. (2010) found that individuals in the social motivation condition had higher PM performance, they focused on event-based prospective memory, which is not directly comparable with the findings of Altgassen et al. This study’s major purpose was to determine whether different intensities of social motivation have different effects on TBPM.

### 1.4. The Present Study

The present study aimed to investigate the effects of social motivation of different intensities on TBPM. According to the motivational cognitive model, individuals will allocate more attention to prospective memory tasks under the high social motivation condition, which is beneficial for the prospective memory tasks to be fully processed. Therefore, we hypothesized that there was no difference in the accuracy of TBPM between the low-intensity social motivation group and the control group, but the accuracy of TBPM for the high-intensity social motivation group was higher than that of the control group. At the same time, compared to the control group, the high-intensity social motivation group had lower accuracy in ongoing tasks and spent more time monitoring times.

## 2. Method

### 2.1. Participants

We recruited 99 participants aged between 18 and 25. The participants were recruited on campus by distributing recruitment advertisements. All participants were students from Henan University in Kaifeng City, Henan Province, China. The experimental testing period was between 20 February and 15 March 2025. When recruiting the participants, we had the following requirements: (1) normal vision or corrected vision; (2) no color blindness; and (3) no participation in memory-related experiments in the past three months. According to the registration order, participants were numbered. The first 33 participants were assigned to the control group, the middle 33 participants were assigned to the low social motivation group, and the last 33 participants were assigned to the high social motivation group. Excluding participants who completely forgot the prospective memory task after the experiment, there were ultimately 92 valid participants remaining in the three groups: the control group (*N* = 29, *M*_age_ = 19.66, *SD* = 1.32, *N*_female_ = 19), the low social motivation group (*N* = 32, *M*_age_ = 9.78, *SD* = 1.21, *N*_female_ = 21), and the high social motivation group (*N* = 31, *M*_age_ = 19.94, *SD* = 1.39, *N*_female_ = 21). After being informed of the experimental requirements, each participant was required to sign an informed consent form. They took the test individually and were paid 20 CNY (about 3 U.S. dollars) after completing the experiment. All participants were required to sign an informed consent form before participating in the experiment. These studies, involving human participants, were reviewed and approved by the Institutional Review Board of Henan Provincial Key Laboratory of Psychology and Behavior (20241205033).

### 2.2. Tasks and Design

A single-factor experimental design between subjects was used in this study, which was divided into a low social motivation group, a control group, and a high social motivation group.

### 2.3. Procedure

Participants were required to read the detailed ongoing task instructions at the beginning of the experiment. After they understood the task requirements, they were required to practice 20 ongoing tasks. Each ongoing task began with the “+” fixation (lasting for 250 ms), followed by an English capital letter in the same place. Participants were required to judge whether it was the same as the preceding letter. They were required to press the “N” key if the two letters were the same or the “M” key if they were not. When the participants responded, the letters disappeared. If they did not respond, the letter disappeared after 1500 ms. Finally, a blank screen appeared for 250 ms. Participants were only permitted to participate in the formal experiment if their ongoing task performance exceeded 0.7 in the practice session; otherwise, they were asked to continue practicing. Participants were informed at the start of the formal experiment that a TBPM task needed to be executed simultaneously with the ongoing task. Participants had to press the “0” key for one minute. By pressing the space bar while performing the TBPM task, participants could check the time at any time, and a time reminder lasting 1 s was given on the screen. The low social motivation group was informed that the experiment was a preliminary study being conducted in preparation for a future experiment and the TBPM task was the focus of the experimenter. In the high social motivation group, the experimenter instructed the participants that the TBPM task was the experimenter’s focus for their graduation thesis, and their successful completion of the task would determine whether the experimenter could graduate successfully. The program was composed of four TBPM tasks for all conditions. Every 68 s, the procedure would allow the participants to take a break, and the next TBPM task could be started by pressing the “P” key after the break. The timing of each TBPM task started at 0:00 and the entire procedure consisted of 132 ongoing tasks and three breaks. Finally, participants were also required to answer two questions following the experiment: (1) they were asked whether they had adopted any strategies to complete the TBPM task and (2) they were asked how important they perceived the TBPM task to be using a 1–9 scale, with 1 being not at all important and 9 being very important (the control group was not asked this question).

## 3. Results

### 3.1. The Accuracy of TBPM

**Figure 1:**
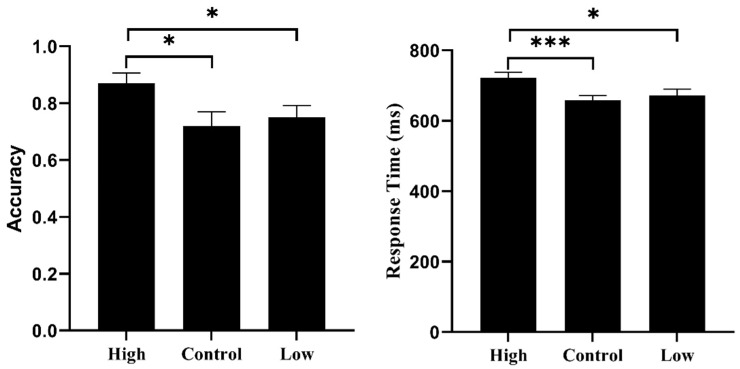
The TBPM task accuracy (**left**) and response time of ongoing task (**right**). Notes: * *p* < 0.05; *** *p* < 0.001; High = high social motivation group; Low = low social motivation group; Control = control group.

If a participant pressed the “0” key within a 5 s deviation, we assumed that they performed the TBPM task correctly. The results of an Analysis of Variance (ANOVA) showed that TBPM accuracy was significantly different for the three groups, *F* (2, 89) = 3.65, *p* < 0.05, η_p_^2^ = 0.08. Post hoc tests indicated that the high social motivation group had higher TBPM accuracy than both the low social motivation group and the control group, *ps <* 0.05. However, there was no difference in the accuracy of the low social motivation group and the control group (*p* > 0.05) (see Figure 1 and Table 1).

### 3.2. The Accuracy of Ongoing Tasks

The ANOVA results showed that there was no significant difference between the three groups in the ongoing tasks’ accuracy, *p* > 0.05 (see Table 2).

### 3.3. The Response Time of Ongoing Tasks

The results of the ANOVA showed that there were significant differences among the three groups in task response time, *F* (2, 89) = 4.26, *p* < 0.05, η_p_^2^ = 0.09. Post hoc tests showed that the response time of the high social motivation group was significantly slower than both the low-social motivation group (*p* < 0.05) and the control group (*p* < 0.001), while there was no difference between the low social motivation group and the control group (*p* > 0.05) (see Figure 1 and Table 1).

### 3.4. The Time Monitoring Frequency

The results of the ANOVA indicated a significant difference in time monitoring frequency among the three groups, *F* (2, 89) = 5.31, *p* < 0.01, η_p_^2^ = 0.11. The data indicated a significant difference (*p* < 0.01) in time checking, revealing that the high social motivation group had a greater frequency than the control group (see Table 2).

### 3.5. The Time Difference of Time Monitoring

To analyze participants’ checking time, we also analyzed the time difference, which refers to the difference between the average time point of time-checking and 1 min, which can indicate how far from the target time participants checked the time. The findings of the ANOVA revealed that the time difference between the three groups was significant, *F* (2, 89) = 9.86, *p* < 0.001, η_p_^2^ = 0.18, and the results of post-hoc tests showed that the high social motivation group had lower time differences than both the low-social motivation group and the control group (*p* < 0.001). There was, however, no difference between the low social motivation group and the control group in this measure (*p* > 0.05).

### 3.6. Number of Strategies

The results of the ANOVA showed that there was no significant difference in the number of strategies among the three groups, *p* > 0.05 (see Table 2).

### 3.7. TBPM Task Importance

Since the control group was not required to report the importance of the TBPM task, task importance was compared between the low and high social motivation groups only. The results of the one-way ANOVA showed that the importance of TBPM tasks reported by the low social motivation group was significantly lower than that reported by the high social motivation group, *F* (1, 61) = 63.94, *p* < 0.001, η_p_^2^ = 0.51 (see Table 2).

## 4. Discussion

This study explores the effects of different intensities (high and low) of social motivation on TBPM. Altgassen et al. (2010) found no promotional effect of social motivation on TBPM among young people. We speculated that, because they directed social motivation towards the ongoing tasks, the level of motivation that was produced in the study was insufficient, and they were unable to determine how social motivation promoted TBPM. To examine how social motivation affects TBPM more thoroughly, we directed social motivation to the TBPM task only. In addition, we changed the motivational intensity to test whether the promotion of social motivation on TBPM was influenced by the intensity of motivation.

The motivational cognitive model posits that PM tasks related to motivation will be perceived as more important, and higher intensity motivation will induce higher task importance (Penningroth & Scott, 2013a). This study found that individuals with high social motivation reported higher importance on TBPM tasks than those with low motivation, suggesting that this study was effective in inducing the requisite intensity of social motivation. The results of TBPM task accuracy showed that participants in the high social motivation condition performed best on the tasks. However, there was no difference between the groups with low social motivation and the control group, indicating that social motivation can promote TBPM, but this promotion can only be produced under the high social motivation condition. This finding is consistent with the current study’s hypothesis.

Why might social motivation promote TBPM performance? The motivational cognitive model suggests that social motivation can promote TBPM in two ways. The first is through the path of controlled processing: individuals allocate more attention resources to motivation-related TBPM tasks, thereby improving TBPM performance (Penningroth et al., 2011). Attention can be divided into internal attention and external attention (Guo et al., 2022; Hu & Qi, 2018). In terms of internal attention, the high social motivation group had the slowest task response speed, indicating that the high social motivation group slowed the task execution speed, which is equivalent to providing more attention resources for TBPM task processing. This was consistent with the prediction of the motivational cognitive model. Unlike the reduction in the ongoing task’s accuracy, slowing the response speed of ongoing tasks is a strategy that indicates that individuals reserve more attention resources for the TBPM task (Anderson et al., 2019). Adequate attention is conducive to more accurate estimation of time intervals (Taatgen et al., 2007), and the better execution of TBPM depends critically on precise time estimation (Gan & Guo, 2019). However, the ongoing task performance of the low social motivation group was not worse, suggesting that low-intensity social motivation did not increase people’s internal attention level and did not improve TBPM performance.

Regarding external attention, we found that the high social motivation group had increased levels of external attention because they checked the time more frequently. However, we found no difference in time monitoring frequency between the two motivation groups, but individuals with high motivation had better TBPM performance. This indicates that the advantage of high motivation in TBPM was not due to external attention. We also discovered that the time difference of the high motivation individuals was the least, which indicated that the time monitoring behavior of high motivation individuals is closer to the destination time. The time difference is closely related to individuals’ internal attention, and individuals always estimate time conservatively and then look to it for feedback (Einstein et al., 1995; Guo et al., 2022). More internal attention will improve the accuracy of time estimation when processing TBPM tasks (Gan & Guo, 2019; Taatgen et al., 2007). This study also found that the internal attention consumption of the high social motivation group was greatest and the time difference was less, which indirectly verified that the internal attention consumption of the high social motivation group also improved the effectiveness of their external attention.

Due to the ease and efficiency of external attention, people are more likely to use it when processing TBPM tasks to guarantee that the work is completed successfully (Reese-Melancon et al., 2019). In this study, however, we found evidence that the high social motivation group relied on internal attention more, which is inconsistent with the findings of previous studies and real-life experience. This may be because TBPM tasks in our daily lives usually have long intervals, for example, ‘I plan to go to the school sports stadium to watch a basketball game at 3 p.m. tomorrow afternoon’. Such a long-time interval TBPM task is difficult to complete through continuous attention, and a greater reliance on external time information is the key to ensure the completion of long-time interval TBPM tasks (Guo et al., 2022). However, when the time interval of the TBPM task is relatively short, as in the present study (1 min only), individuals are more likely to successfully complete the task by using continuous internal attention. The shorter time interval may lead to a greater dependence on internal attention for promoting TBPM in the high social motivation group.

The motivational cognitive model holds that there may also be an automated pathway for social motivation to promote TBPM. This approach suggests that individuals in the social motivation condition will adopt more strategies to make prospective memory tasks easier to maintain and extract, increasing their TBPM performance as a result. In this study, we did not find that the high social motivation group adopted more strategies, which is inconsistent with the predictions of the motivational cognitive model. However, we asked participants to self-report the strategies adopted, and this subjective indicator may not be sufficiently valid. For example, some participants believed that strategic time monitoring was a strategy, while others believed it was not and therefore failed to report it. According to the motivational cognition model, individuals could adopt some effective strategies in advance under motivational conditions, which was conducive to the monitoring of prospective memory cues and the extraction of intended content. However, the use of strategies may also consume attention resources. For example, some participants use strategies such as counting and beating to improve the accuracy of time estimation in TBPM task processing, but these strategies also consume additional attention. Even though some participants use exercises and other techniques to reduce the dependence of TBPM tasks on attention, these strategies themselves require effort in advance (Ihle et al., 2018; Guo et al., 2019). Therefore, the use of a strategy does not always work in the case of automated processing. The automated processing path predicted by the motivational cognitive model is relatively limited, and for the sake of validity, we will not draw definite conclusions regarding the use of strategies.

Although this study has revealed the influence of social motivation intensity on TBPM to a degree and validates social motivation as a mechanism to promote TBPM within the framework of the motivational cognitive model, our findings also have certain limitations. First, we adopted TBPM tasks with relatively short time intervals, which require continuous attention to guarantee successful task completion (Guo et al., 2022). However, TBPM tasks in daily life often occur at intervals of several hours or even days, and those tasks cannot be completed through continuous attention. The promotional effect of social motivation on long-term TBPM may differ from the findings of the present study. Second, this study reflects changes in attention through a limited number of behavioral responses. In comparison, eye-tracking technology can more accurately measure the objects of attention, attention load, and the trajectory of attention, which comprehensively reflects changes in attention. Finally, in this study, instructions were used in the laboratory to induce social motivation, but their ecological validity was relatively low. Future research could consider inducing social motivation in real life by setting up some social interaction scenarios. Finally, in this study, instructions were used in the laboratory to induce social motivation, but their ecological validity was relatively low. Future research could consider inducing social motivation in real life by setting up some social interaction scenarios.

## 5. Conclusions

This study focused on the effects of different intensities of social motivation on TBPM and found that only high social motivation promoted TBPM performance. Both increased internal and external attention contributed to the TBPM performance of the high social motivation group, consistent with the predictions of the motivational cognitive model. The finding that the high social motivation group had better TBPM performance compared to the low social motivation group, but without the consumption of additional external attention, suggests that social motivation’s promotion of TBPM does not depend on external attention. The findings also showed that the effect of social motivation on TBPM promotion was independent of additional external strategies, which was not consistent with the motivational cognitive model’s prediction. The conclusions of this study have several theoretical implications. On the one hand, we explored the influence of social motivation on TBPM within the framework of the motivational cognitive model and verified the applicability of the framework. On the other hand, we explored the changes in attention with high and low levels of social motivation in promoting TBPM, further extending the understanding of the framework of the motivational cognitive model.

## Figures and Tables

**Table 1:** TBPM accuracy and ongoing task performance of the three groups (*M* ± *SD*).

	Accuracy of TBPM	Accuracy of Ongoing Task	Response Time of Ongoing Task (milliseconds)
High	0.87 (0.20)	0.85 (0.03)	722 (90)
Control	0.72 (0.27)	0.85 (0.06)	658 (75)
Low	0.75 (0.23)	0.83 (0.03)	672 (101)

Notes: High = high social motivation group; Low = low social motivation group; Control = control group.

**Table 2:** Time monitoring indicators and subjective reporting indicators for the three groups (*M* ± *SD*).

	Number of Viewing Time	Time Difference (s)	Strategy	Importance
High	3.46 (1.13)	9.78 (3.84)	0.81 (0.70)	8.52 (0.57)
Control	2.59 (1.22)	13.76 (4.18)	0.52 (0.69)	
Low	2.99 (0.73)	12.64 (2.67)	0.66 (0.79)	6.78 (1.07)

Notes: High = high social motivation group; Low = low social motivation group; Control = control group.

## Data Availability

The data that support the findings of this study are available from the corresponding author upon request.
